# Voluminous Extracardiac Adult Rhabdomyoma of the Neck: A Case Presentation

**DOI:** 10.1155/2012/984789

**Published:** 2012-12-06

**Authors:** Riccardo Maglio, Scicchitano Francesco, Magistri Paolo, Valabrega Stefano, D'Angelo Francesco, Ramacciato Giovanni

**Affiliations:** General Surgery Department, S. Andrea Hospital, Sapienza University of Rome, Grottarossa Street 1035-1039, 00189 Rome, Italy

## Abstract

*Introduction*. Rhabdomyomas of the head and neck are exceptionally rare benign mesenchymal tumors. Rare cases have been reported to involve other sites of the body including the head and neck regions. *Case Presentation*. We report a case of voluminous extracardiac adult rhabdomyoma affecting adult patients and initially seen as slowly growing, indolent neoplasms. The patient is a seventy-year old male Italian patient. *Conclusion*. Adult extracardiac rhabdomyoma is a rare benign tumor that may present with symptoms that vary from aerodigestive tract obstruction to remaining asymptomatic for many years. Although histology is very characteristic, several differential diagnoses have to be considered. To our knowledge, this is the first case of voluminous adult-type symptomless rhabdomyoma.

## 1. Introduction

Rhabdomyomas are benign mesenchymal tumours with skeletal muscle differentiation. They can be divided into extracardiac-and intracardiac-type depending on the localization. The extracardiac type is extremely rare, with a propensity for occurrence in the head and the neck and for multiple localization (3–10%, synchronous or asynchronous); recurrences may be observed after complete excision (10 to 40%). They are virtually never associated with other malformations; on the other hand intracardiac rhabdomyomas are often associated with tuberous sclerosis (up to 86% of patients with TS in series with echocardiographical evaluation) or other genetical disorders.

Extracardiac rhabdomyomas can be divided into four clinically and morphologically different types: the adult-(AR), the fetal-(FR), the genital-type (GR), and rhabdomyomatous mesenchymal hamartoma. AR mainly affects elder people, with a mean age of occurrence of 60 years, with a 3 : 1 male : female ratio. Malignant transformation has not been described [1]. Histologically the AR presents polygonal cells, eosinophilic granular cytoplasm, cross-striations, and spider cells; the expression of desmin, muscle-specific actin, myoglobin, and myogenin can be immunohistochemically demonstrated [1]. The most important differential diagnoses are granular cell tumors, hibernomas, oncocytomas, and paragangliomas, but all these tumors do not histologically present cross-striation and do not content glycogen [2]. FR is rarer than AR; it is histologically characterized by small eosinophilic cells, sometimes with cross-striations, rare nucleoli, and the same immunohistochemical features of AR [1]. Intracardiac rhabdomyomas are the most common tumours in the pediatric age group. They are also the tumours most commonly diagnosed during the prenatal period by foetal echocardiography. They may obstruct valvular orifices or occludes intracavitary spaces, leading to respiratory distress and congestive heart failure [3]. Some authors argue that intracardiac rhabdomyomas may be due to delay or failure of apoptosis which occurs as part of the normal remodeling process in the heart [4]. They can be single or multiple, well circumscribed, noncapsulated nodules which may vary in size from millimeters to several centimeters. Histologically there is a strong reaction with PAS reagent, reflecting the presence of abundant intracellular glycogen, while immunohistochemical studies demonstrate the presence of myoglobin, desmin, actin, and vimentin. Tumour cells do not express cell proliferation markers such as Ki-67 and PCNA, indicating that the lesions are more likely hamartomas than neoplasms. They may spontaneously regress, but severe symptoms may precipitate the need for surgical excision. Antiarrhythmic drugs can be helpful if arrhythmias are the presenting symptom [3].

## 2. Case Report

**Figure 1:**
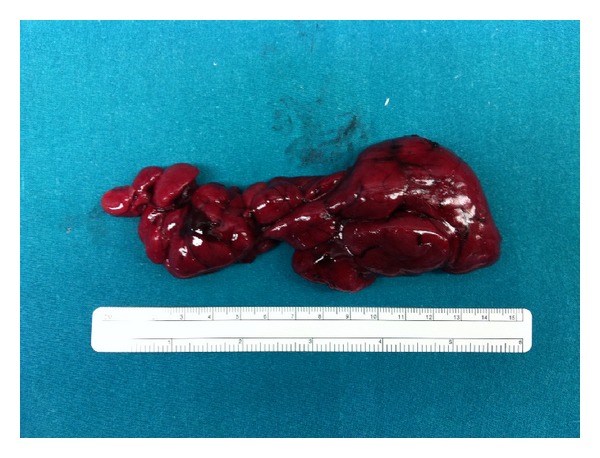
Adult rhabdomyoma (the large tumor was red lobulated mass).

**Figure 2:**
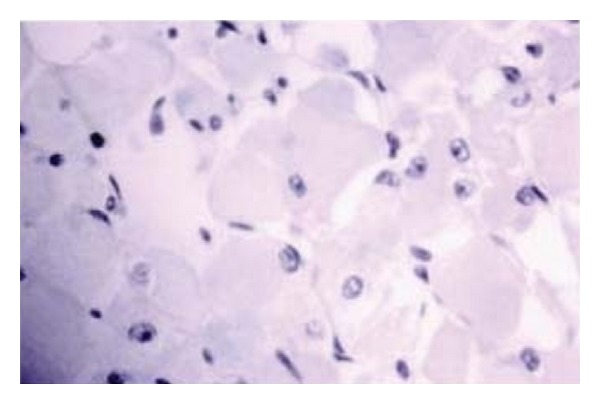
Skeletal muscle (it shows cytoplasmic positivity for MSA, desmin).

A seventy-year old male patient with hypertension and asthma comorbidity came to our attention with a 8-9 cm mass in the left submandibular region. He had a history of alcohol abuse, and he had a smoking history of two to three packs of cigarettes per day for approximately 60 years. This mass appeared one year before, slowly growing, giving no pain to the patient. A cervical ultrasound was performed and described an oval hypoechoic mass which did not infiltrate the thyroid or the submandibular gland. Even a CT scan was performed. An aspiration biopsy from the tumor showed a smear of low cellularity dominated by large polygonal to round cells with abundant acidophilic granular cytoplasm. Chest radiographs showed no acute pulmonary process. The patient underwent a surgical excision of the mass via an open transcervical approach. During the procedure, the jugular vein seemed compressed and shifted forward by the mass which was in contact with the hyoid bone and with the upper pole of the left thyroid but did not infiltrate these structures. A 7 mm jp drainage was applied at the end of the procedure. The tumor was then sent to pathology: macroscopically it was a brown and brick red lobulated mass, was soft in consistency, and measured 8.5 × 2.5 × 1.5 cm (Figure 1). Immunohistochemical staining of the cervical lesion showed diffuse cytoplasmic positivity for myoglobin and desmin in most of the cells with a few pale staining cells. The histopathology was consistent with adult extracardiac rhabdomyoma (Figure 2).

## 3. Discussion

Extracardiac rhabdomyomas are very rare tumors comprising less than 2% of the neoplasm of the striated origin. This entity was first described in 1897 by Pendl, who presented a case of fetal type [4], and fewer than 100 cases have been reported in the literature. Extracardiac rhabdomyomas prefer the head and the neck regions, arising from the musculature of the third and fourth branchial arches. Most sites located in the head and neck regions include the larynx [5], pharynx [6–8] strap muscles [5], sternocleidomastoid [5], parapharyngeal [9, 10] and submandibular [6, 9, 10] spaces, floor of the mouth, tongue and base of the tongue [8], cheeks, lips, and eyelids. Rarely, rhabdomyomas have been found to be located in the extremities, esophagus, stomach, mediastinum, orbit, prostate, and heart [11]. Symptoms, which are nonspecific, depend on the localization and the size of the tumor [2]. Occasionally, when lesions involve the oral cavity and oral pharynx, a mass in the throat may be observed. In other cases, symptoms of dysphagia, hoarseness, or respiratory distress may be reported when the lesions involve the aerodigestive tract. Lesions involving the neck region and submandibular region may present as neck masses. Numerous diseases have been mentioned with the differential diagnosis of AR such as hibernoma, oncocytoma, paraganglioma, reticulohistiocytoma, lymphoma, rhabdomyosarcoma, and crystal-storing histiocytosis. Among these hibernomas are the most relevant soft tissue lesions and, therefore, are especially considered. The gold standard treatment is surgery. It can recur, but it never turns malignant. Recurrence may be as high as 42% 10, and there has been one case with three recurrences in a 35-year period [11]. This is probably due to incomplete excision or multifocality which is seen in 14–26% of the cases [11]. In this particular case, a complete resection was believed to have been performed at the time of initial surgery. However, we suspect that a microscopic component must have remained, which led to recurrence of the rhabdomyoma. The immunohistochemical features of rhabdomyoma are identical to those of normal skeletal muscle cells because it shows cytoplasmic positivity for MSA (muscle-specific actin), desmin. Rhabdomyomas have been reported in all age groups, but Golz indicates that the mean age of occurrence for adult extracardiac rhabdomyoma is 52.2 years [12]; these tumors are more frequent in males as reported in a series by Gajda et al. [13].

## 4. Conclusion

Adult extracardiac rhabdomyoma is a rare benign tumor that may present with symptoms that vary from aerodigestive tract obstruction to remaining asymptomatic for many years until incidentally being diagnosed in surgery or post-mortem. It is most commonly found in the head and neck regions and should be considered in the differential diagnosis of masses in this region.

## Abbreviations

AR: Adult rhabdomyoma
FR: Fetal rhabdomyoma
GR: Genital rhabdomyoma
MSA: Muscle-specific actin.
